# SARS-CoV-2 and Subacute Thyroiditis: A Case Report and Literature Review

**DOI:** 10.1155/2022/6013523

**Published:** 2022-06-30

**Authors:** Amir Mohammad Salehi, Hossain Salehi, Hossein Ali Mohammadi, Jamileh Afsar

**Affiliations:** ^1^Student of Medicine, School of Medicine, Hamadan University of Medical Sciences, Hamadan, Iran; ^2^Gastroenterology Ward, Baharlo Hospital, Tehran University of Medical Sciences, Tehran, Iran; ^3^Student Research Committee, Hamadan University of Medical Sciences School of Medicine, Hamadan, Iran; ^4^Clinical Research Development Unit of Shahid Beheshti Hospital, Hamadan University of Medical Science, Hamadan, Iran

## Abstract

**Introduction:**

Subacute thyroiditis (SAT) is an inflammatory disorder of the thyroid gland, usually triggered by a recent viral or bacterial infection of upper respiratory tracts. The disease is characterized by neck pain radiating to the ears and thyroid gland tenderness. In most cases, it is associated with a transient episode of hyperthyroidism, which is followed by euthyroidism. However, sometimes, it manifests itself with hypothyroidism. *Case Presentation*. The present report described a case of SAT who was a 55-year-old man presenting to an endocrine clinic with tachycardia, tremor, and neck pain radiating to the jaw and ears. His thyroid function test revealed thyrotoxicosis, and thyroid ultrasound findings were consistent with SAT. The patient reported a history of COVID-19 about 15 days before presentation, which was confirmed by a positive PCR test for SARS-CoV-2.

**Conclusions:**

It is of great importance for physicians to note that thyrotoxicosis in a patient with a recent history of COVID-19 can be due to SAT. Therefore, they should not begin antithyroid drugs without ordering proper investigations.

## 1. Introduction

In December 2019, the World Health Organization (WHO) was notified of a case of pneumonia with unknown etiology in Wuhan, China. Investigations on this case by the Chinese scientists led to the discovery of the severe acute respiratory syndrome coronavirus 2 (SARS-CoV-2). On March 11, 2020, the mentioned disease, which was named as the coronavirus disease 2019 (COVID-19), was declared a pandemic by the WHO. According to the mentioned organization, 254,256,432 cases of infection, as well as 5,112,461 cases of related mortality, had been reported until November 17, 2021 [1]. The first clinical findings reported included respiratory symptoms associated with bilateral pulmonary ground-glass lesions on CT scan and radiography [2]. Since then, some extrapulmonary manifestations have also been reported as early disease manifestations or related complications, including gastrointestinal, hepatic, biliary, pancreatic, cardiovascular, ophthalmologic, and neurological manifestations [3, 4]. Until March 26, 2021, twenty-two cases of subacute thyroiditis (SAT) were reported that were potentially due to concomitant or recent COVID-19 (Table 1). However, there were no reports of any associations between the coronavirus infections and SAT before the current pandemic [20]. The present study reported a case of SAT after recent COVID-19.

## 2. Case Presentation

**Figure 1:**
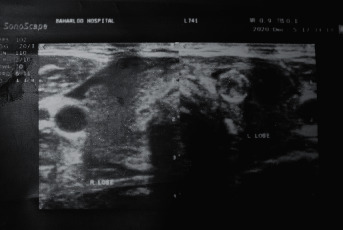
Thyroid ultrasound findings.

A 55-year-old man living in Tehran, Iran, presented to the emergency department with complaints of diffuse neck pain extending to the jaw, severe and frequent coughs, and hot flashes. On clinical examination, the thyroid gland was tender, while the vital signs were normal. The patient reported a recent COVID-19 diagnosed with a positive PCR test for SARS-CoV-2 about 15 days before presentation, for which he was treated with favipiravir and corticosteroids (AMP dexamethasone 8 mg daily for 3 days). A thyroid function test (TFT) with the CLIA method was ordered for the patient, which showed elevated T3 and T4 and decreased TSH (Table 2). The cell blood count (CBC) results were as follows: WBC, 10.4 × 10^9^/l (normal range: 4–10 × 10^3^/mm^3^); RBC, 3.95 × 10^12^/l (normal range: 4.6–6.2 10^12^/l); Hb, 11.9 g/dL (normal range: 13–17 g/dL); HCT, 34.5% (normal range: 39–50%); PLT, 432 × 10^9^/l (normal range: 140–400 10^9^/l); and lymphocyte rate, 19% (normal range: 20–45%). Also, the following inflammatory markers were elevated as well: ESR, 121 mm/h (normal range: up to 20 mm/h) and CRP titer, 92.6 mg/l (normal range: <10 mg/l). The thyroid ultrasound revealed a mild diffuse goiter with a focal and ill-defined hypoechoic area, which is a characteristic finding of SAT (Figure 1). The patient denied any history of thyroid diseases or chronic medication use. Stasiak and Lewiński [21] proposed new diagnostic criteria for COVID-19-induced SAT based on the new characteristics of SAT triggered by SARS-CoV-2. We compared the abovementioned criteria (Table 3) with the clinical findings of our patient, finding that the present case met the criteria for COVID-19-induced SAT, so the patient was diagnosed with SAT induced by COVID-19, for which he received aspirin as the first-line treatment. Following 10 days of treatment, no improvement was observed, and the patient complained of front neck pain while talking. Therefore, prednisolone 25 mg/d was prescribed for the patient. Following three weeks of prednisolone treatment, the symptoms were resolved, and thyroid function tests repeated for the patient indicated that T3, T4, and TSH levels returned to their normal ranges.

## 3. Discussion

The first report of COVID-19-induced SAT was an 18-year-old woman with symptoms consistent with SAT that was initiated 15 days after an episode of mild COVID-19 diagnosed by a positive PCR test for SARS-CoV-2 at that time. The primary symptoms of this patient were the typical symptoms of SAT, including low-grade fever, neck pain, fatigue, and palpitations. Laboratory tests ordered were consistent with SAT and included accelerated ESR, elevated CRP, and thyrotoxicosis in the thyroid function test. Patients underwent corticosteroid therapy, leading to symptom resolution in a few days [7]. After the publication of the mentioned report, the 2-week interval between COVID-19 and SAT was considered to be relatively short. However, other case reports and case series studies published afterward showed that surprisingly, SAT symptoms could initiate shortly after the beginning of COVID-19. According to the observations, SAT symptoms may develop concomitant with COVID-19 or shortly after, with a maximum interval of 6 weeks [9, 10] (Table 1). Although neck pain might be attributed to a viral infection, so it is usually underreported, and the prevalence of painless COVID-19-induced SAT is increasing [9, 21–24]. Thus, it seems that neck pain, which was a symptom previously regarded as the key diagnostic criterion, is not always present. However, in our search, only one case of painless SAT was reported as a “case report” [7]. Our patient had a complaint of neck pain. In fact, 3 groups of patients may develop painless COVID-19-induced SAT. The first group includes the patients with COVID-19 who are taking NSAIDs or analgesics due to their COVID-19-related symptoms. Therefore, they may not feel any neck pain due to the analgesic effects of these medications. The second group includes the ICU-admitted patients with COVID-19-induced SAT who cannot feel or express pain due to their condition or strong medications [22]. Finally, the third group includes patients with true, painless COVID-19-induced SAT.

Since asymptomatic SARS-CoV-2 infection is highly prevalent, it is recommended to order PCR tests for all patients presenting with SAT symptoms. This painless course may be related to reduced lymphocytic-plasmacytic infiltration in the thyroid gland due to lymphopenia present in COVID-19 patients [25]. In general, few patients with SAT develop the signs and symptoms of thyrotoxicosis. However, a sudden onset of tachycardia, the deterioration of previous tachycardia, and the onset of arrhythmias are the typical symptoms of COVID-19-induced SAT [11, 19]. Thyroid function tests in SAT patients with severe COVID-19 have shown thyrotoxicosis with elevated FT4 and reduced TSH and FT3, which corresponds to the simultaneous presence of SAT and nonthyroidal illness syndrome during the course of severe systemic disease [26].

The development of thyroid diseases in the course of severe acute respiratory syndrome (SARS) has been associated with various mechanisms of thyroid damage, including excessive immune response, the immunodeficiency associated with infection, or direct cellular damage [25, 27]. SARS-CoV-2 exhibits significant tissue tropism, including high affinity to the thyroid tissue. The key factor in SARS-CoV-2 infection is the angiotensin-converting enzyme 2 (ACE-2) receptor, which enables viral entry. Thyroid cells are rich in ACE-2 [21, 23, 24, 28, 29]. According to Rotondi et al., the mRNA encoding the ACE-2 receptor is highly expressed in thyroid follicular cells, making them a potential target for SARS-CoV-2 [30]. The highest incidence of SAT has been reported in middle-aged women, and the females account for 75–80% of all SAT cases [26, 31]. However, the symptoms suggestive of SAT are present in 10% and 20% of ICU-admitted patients with COVID-19 and those hospitalized in non-ICU wards due to this disease, respectively [22, 23]. Moreover, some pediatric cases of SAT have been reported recently [32, 33].

Stasiak et al. showed that the susceptibility to SAT and the chance of recurrent SAT could be related to HLA [34, 35]. They performed a study on Caucasian population, reporting that the risk of recurrence was significantly higher in patients with both HLA-B^∗^18 : 01 and HLA-B^∗^35 [34]. Also, they demonstrated that even the ultrasound characteristics of SAT thyroid lesions were related to HLA, with HLA-B^∗^18 : 01 being the determining factor [26]. According to their findings, multiple hypoechoic blurred lesions typically observed in SAT were rarely found in patients positive for HLA-B^∗^18 : 01. These patients had different ultrasound patterns, which were mostly observed in patients with only HLA-B^∗^18 : 01, without any haplotype correlated with SAT. Most of these patients had a unilateral and single lesion that was homogenously hypoechoic and had filled the whole affected lobe, mimicking a large thyroid nodule. However, in patients with both HLA-B^∗^18 : 01 and HLA-B^∗^35, the main difference from the typical pattern was related to the shape of SAT lesions, which were patchy or round, imitating actual thyroid nodules [26]. It is worth mentioning that we did not perform any HLA testing for our patient.

Regardless of proper diagnosis and treatment, the chance of SAT recurrence is relatively high and varies between studies from a few to over 20% [34, 36]. These controversies are probably due to different study populations (Caucasian vs. Asian). Recurrences of SAT can occur either soon after the treatment completion or after a significant time interval, sometimes many years from the first episode [34]. Until recently, the cause of SAT recurrences was unknown. The only findings were that excessively fast tapering of corticosteroids was one of the causes [37]. As a matter of fact, the differential diagnosis of SAT is challenging and necessitates ruling out the false negative and false positive SAT cases due to disease mismanagement or any serious undiagnosed conditions, such as covert malignancies. Since hospitalized patients usually receive corticosteroids as the COVID-19 treatment protocol, treating any undiagnosed SAT in these patients does not often result in serious problems. However, the misdiagnosis of hyperthyroidism in the outpatients with symptoms suggestive of painless SAT might be challenging, and any misuse of antithyroid drugs should be avoided. The other important issue is that SAT can be the only sign of COVID-19. So, patients with SAT should be tested for COVID-19 to prevent disease spread.

According to other studies, 24 cases of concomitant or post-COVID-19 subacute thyroiditis were reported until March 26, 2021. If we consider the present study, 18 of these patients were female, while 7 were male. Subacute thyroiditis is a disease which is more common in women than men, but our case was male. The age range of the male patients was 34–58 years, while it was 18–69 years for women. Our patient's age was in the age range of the mentioned patients.

The duration between COVID-19 infection and the onset of subacute thyroiditis was 1–6 weeks, including one week for 1 case, two weeks for 4 cases (include our case), three weeks for 1 case, four weeks for 13 cases, five weeks for 1 case, and six weeks for 2 cases. Concomitant onset was observed in 4 patients. Our patient's previous infection with COVID-19 was confirmed by PCR, but in 10 cases, there was no mention of the previous PCR test for COVID-19 confirmation, and just the clinical findings and high-risk occupation and communications of patients were considered. Of these 10, 8 cases had serological tests indicated past infection with COVID-19. Thyroid function tests (TFT) showed thyrotoxicosis in all patients, including our patient, except for one case in which TFT was not available. In terms of ultrasound findings, they supported subacute thyroiditis in all patients (including presented case) except for 2 cases that ultrasound was not available. All patients treated with NSAIDs or corticosteroids or both responded well to the treatment (Table 1).

## Figures and Tables

**Table 1:** Review of previous post-COVID SAT.

	Age/sex	Time between positive COVID-19 PCR and onset of SAT symptoms	Clinical features	COVID-19 PCR testing on admission	Inflammatory markers	TFT on admission	Findings of thyroid USG	Treatment	Reference
Patient 1	55/male	15 days	A painful tender thyroid gland with pain radiating to the jaw, fever, tachycardia severe and frequent cough, and flushing	Not available	ESR = 121 mm/h CRP = 92.6 mg/l	Thyrotoxic	Complete heterogeneous echotexture suggestive of goiter was observed in the right thyroid lobe with ring calcification in the superior part of the left lobe	(1) Aspirin (discontinued after one week) (2) Prednisolone after aspirin	Current case
Patient 2	56/male	28 days	Anterior neck pain, myalgia, and fatigue	Not available	ESR = 78 mm/h CRP = 45 mg/l	Thyrotoxic TSH (*µ*IU/mL) = 0.139 Free T4 (ng/dl) = 1.39	Heterogenous thyroid, hypoechoic areas, decreased vascularity (unilateral, right lobe)	Naproxen sodium (NSAID)	[5]
Patient 3	38/female	28 days	Anterior neck pain, myalgia, fatigue, back pain, and headache	Not available	ESR = 68 mm/h CRP = 18.4 mg/l	Thyrotoxic TSH (*µ*IU/mL) = 0.99 Free T4 (ng/dl) = 1.02	Heterogenous thyroid, hypoechoic areas, decreased vascularity (bilateral)	Naproxen sodium (NSAID)	[5]
Patient 4	41/female	28 days	Tender neck swelling, fevers, neck pain, odynophagia, fatigue, hand tremors, and palpitations	(−)	CRP = 36.4 mg/l ESR = 107 mm/h	Thyrotoxic normal T3up TPOAb (+) TSI (−) TSHrAb (−)	(1) Heterogenous thyroid gland (2) Bilateral patchy ill-defined hypoechoic areas	(1) Ibuprofen 600 mg PO q6h (2) Prednisone 40 mg/d PO (followed by taper)	[6]
Patient 5	18/female	19 days	Anterior neck pain radiating to the jaw, fatigue, fevers, and palpitations	(−)	ESR = 90 mm/h CRP = 69 mg/l	Thyrotoxic/sTg detected (low level) TPOAb (−) TSHrAb (−) TgAb (+)	Multiple diffuse hypoechoic areas	Prednisone 25 mg/d PO (followed by taper)	[7]
Patient 6	41/female	Concurrent illness	A painful tender thyroid gland, fevers, left TMJ tenderness, and pharyngitis	(+)	CRP = 101 mg/l ESR = 134 mm/h	Thyrotoxic TPOAb (−) TSHrAb (−) TgAb (−)	(1) Heterogenous thyroid parenchyma (2) Relative diffuse decrease of vascularity	(1) HCQ 200 mg PO q12 h × 5 days (2) Prednisolone 16 mg/d PO (followed by taper)	[8]
Patient 7	69/female	Concurrent illness	Cough, fever, dyspnea, insomnia, agitation, and palpitations	(+)	Not available	Thyrotoxic High sTg TSHrAb (−) TPOAb (−) TgAb (−)	(1) Enlarged hypoechoic thyroid (2) Decreased vascularity (3) Known 30 mm homogenous nodule in the right lobe (with peripheral vascularization)	(1) HCQ (2) Methimazole(later discontinued) (3) Methylprednisolone IV × 3 days (4) Prednisone 25 mg/d PO (followed by taper)	[9]
Patient 8	43/female	6 weeks	Tenderness anterior neck, fever, tremors, fatigue, and palpitations	Not available	Not available	Thyrotoxic High sTg TPOAb (−) TSHrAb (−) TgAb (−)	Diffusely enlarged and hypoechogenic thyroid gland (thyroid scintigraphy showed markedly reduced 99mTc-pertechnetate uptake)	(1) Prednisone 25 mg/d PO (followed by taper)	[10]
Patient 9	38/female	16 days	Anterior neck pain radiating to the jaw, asthenia, fever, palpitation, and anorexia	(−)	ESR = 47 mm/h CRP = 11.2 mg/l	Thyrotoxic TgAb <30 IU/mL TPOAb <10 IU/mL TRAb <1.5 IU/mL	Enlarged thyroid gland with multiple hypoechoic areas and absent vascularization at color Doppler	Prednisone 25 mg/d (followed by taper)	[11]
Patient 10	29/female	30 days after starting quarantine (a PCR test is not available)	Anterior neck pain radiating to the jaw, asthenia, fever, palpitation, and sweating	Not available (negative at the end of quarantine)	ESR 110 mm/h CRP 7.9 mg/l	Thyrotoxic Tg 80 mg/l TgAb 38 IU/mL TPOAb <10 IU/mL TRAb <1.5 IU/mL	Multiple diffuse hypoechoic areas and low vascularization at color Doppler	(1) Prednisone 25 mg/d (followed by taper) (2) Propranolol 40 mg/d	[11]
Patient 11	29/female	36 days after onset of COVID-19 symptoms (a PCR test is not available)	Anterior neck pain radiating to the jaw, palpitation, and sweating	Not available	Not available	Not available	Diffuse enlarged gland, with multiple hypoechoic areas and absent vascularization at color Doppler	(1) Ibuprofen 600 mg/d (2) Low dose of levothyroxine (after 47 days of admission)	[11]
Patient 12	46/female	29 days	Anterior neck pain radiating to the jaw, asthenia, fever, palpitation, insomnia, anxiety, and weight loss	(−)	CRP = 8 mg/l	Thyrotoxic TRAb <1.5 IU/mL	An enlarged thyroid with multiple hypoechoic areas	Prednisone 25 mg/d	[11]
Patient 13	29/female	6 weeks after COVID-19 infection (a PCR test is not available)	Fever, odynophagia, exertional tachycardia, shortness of breath, weight loss, front neck tenderness, fine bilateral hand tremors, and palpable left thyroid lobe	(−)	CRP = 44 mg/l ESR = 88 mm/h	Thyrotoxic TPOAb (−) TSI (−)	Heterogeneously enlarged thyroid gland	(1) Prednisone 20 mg/d, then 40 mg/d, and then tapered off (2) Atenolol 25 mg/d, then 50 mg/d, and then discontinued	[12]
Patient 14	58/male	Concurrent illness	Anterior neck pain, fever, diffusely enlarged thyroid gland, and tachycardia	(+)	ESR = 110 mm/h CRP = 16.6 mg/l	Thyrotoxic	Diffuse bilateral enlargement of thyroid with hypoechogenicity and increased vascularity on color Doppler and a solitary nodule in each lobe	(1) Combination of analgesics, favipiravir and azithromycin, along with zinc tablets and vitamin C capsules (2) Prednisolone 30 mg/d 9 followed by taper) (3) Propranolol 40 mg/d (4) Levothyroxine 50 *μ*g/day (after one month of admission)	[13]
Patient 15	47/female	Concurrent illness	Anterior neck pain radiating to the right submandibular region	(+)	CRP = 50.9 mg/l	Subclinical hyperthyroidism TPOAb (−) Anti-TGB Ab (−) 69TRAb (−)	Slightly enlarged right thyroid lobe, with ill-defined hypoechogenicity and normal vascularity in both lobes	(1) Mefenamic acid was started, but was later shifted to celecoxib due to epigastric pains. Oral hydroxychloroquine and intravenous ceftriaxone were initiated (2) Oral levothyroxine (after 8 weeks of admission)	[14]
Patient 16	26/female	30 days after COVID-19 infection (a PCR test is not available)	Fever, fatigue, palpitation, painful, tender, and slightly thyroid gland	(−)	ESR = 70 mm/h CRP = 28 mg/l	Thyrotoxic	Bilateral hypoechoic areas in the thyroid gland	Prednisolone 25 mg/d (followed by taper)	[15]
Patient 17	37/female	30 days after COVID-19 infection (a PCR test is not available)	Fever, fatigue, palpitation, painful, tender, and slightly thyroid gland	(−)	ESR = 56 mm/h CRP = 38 mg/l	Thyrotoxic	Bilateral hypoechoic areas in the thyroid gland	Prednisolone 25 mg/d (followed by taper)	[15]
Patient 18	35/male	30 days after COVID-19 infection (a PCR test is not available)	Fever, fatigue, palpitation, painful, tender, and slightly thyroid gland	(−)	ESR = 45 mm/h CRP = 18 mg/l	Thyrotoxic	Bilateral hypoechoic areas in the thyroid gland	Prednisolone 25 mg/d (followed by taper)	[15]
Patient 19	41/female	30 days after COVID-19 infection (a PCR test is not available)	Fever, fatigue, palpitation, painful, tender, and slightly thyroid gland	(−)	ESR = 83 mm/h CRP = 43 mg/l	Thyrotoxic	Bilateral hypoechoic areas in the thyroid gland	Prednisolone 25 mg/d (followed by taper)	[15]
Patient 20	52/male	30 days after COVID-19 infection (a PCR test is not available)	Fever, fatigue, palpitation, painful, tender, and slightly thyroid gland	(−)	ESR = 76 mm/h CRP = 51 mg/l	Thyrotoxic	Bilateral hypoechoic areas in the thyroid gland	Prednisolone 25 mg/d (followed by taper)	[15]
Patient 21	34/female	30 days after COVID-19 infection (a PCR test is not available)	Fever, fatigue, palpitation, painful, tender, and slightly thyroid gland	(−)	ESR = 39 mm/h CRP = 23 mg/l	Thyrotoxic	Bilateral hypoechoic areas in the thyroid gland	Prednisolone 25 mg/d (followed by taper)	[15]
Patient 22	28/female	13 days	Fever, anterior neck pain radiating to the jaw, palpitation, sore throat, and severe asthenia	(−)	ESR = 116 mm/h CRP = 173 mg/l	Thyrotoxic TgAb (−) TPOAb (−) TRAb (−)	Not available (thyroid scintigraphy with 5.73 mCi of 99mTc-pertechnetate was performed on May 26th, which showed absence of uptake in the gland)	(1) Aspirin 500 mg q6h (2) Propranolol 40 mg q6h	[16]
Patient 23	37/female	30 days	Severe neck pain radiating to the right ear and jaw, fatigue, moderately enlarged tender thyroid gland, and neck adenopathies	Not available	ESR = 72 mm/h CRP = 66 mg/l	Thyrotoxic TgAb (−) TPOAb (−)	Not available	Not available	[17]
Patient 24	37/male	30 days after COVID-19 infection (a PCR test is not available)	Anterior neck pain with tenderness, fatigue, chills, palpitation, anorexia, and weight loss	(−)	ESR = 31 mm/h CRP = 14 mg/l	Thyrotoxic TPOAb (−) TSI (−)	Diffusely heterogeneous echotexture	(1) Aspirin (2) Propranolol (3) 1.6 mcg/kg/day of oral levothyroxine 50 days after admission	[18]
Patient 25	34/male	5 days	Anterior neck pain, tachycardia, diffuse asymmetric goiter with tenderness, and few bilateral palpable cervical lymph nodes	(+)	CRP = 122 mg/l	Thyrotoxic TPOAb (−) TRAb (−)	Enlarged thyroid gland with heterogeneous echotexture. Both lobes had hypoechoic areas with ill-defined margins corresponding to the hard regions palpable. Color flow Doppler showed reduced blood flow in both lobes. There were no definite nodules seen in the thyroid gland. A few cervical lymph nodes with normal morphology were seen.	(1) Prednisolone 20 mg/d (followed by taper) (2) Atenolol 25 mg/d	[19]

**Table 2:** TFT at multiple time points during the patient illness.

Test (reference range)	The first visit	One month later
T4 (5.1–14.1 ng/dL)	15.8 ng/dL	8.8 ng/dL
T3 (40–181 ng/dL)	190 ng/dL	98 ng/dL
TSH (0.3–8 mIU/l)	0.29 mIU/l	4.68 mIU/l

**Table 3:** Comparison the mentioned criteria with the clinical findings of our patient.

COVID-19-related SAT criteria	Present in our patient
Main criteria (all should be met)	
Laboratory: elevation of ESR or at least CRP	+
Ultrasound: hypoechoic area/areas with blurred margin and decreased vascularization in US	+

Remarks related to COVID-19 pandemic (should be taken into account during pandemic)	
SAT diagnosis should be considered in patients with/after SARS-CoV-2 infections with	
Unexpected	
De novo presence of tachycardia or arrhythmias	−
Deterioration of previously present tachycardia or arrhythmias	+
Deterioration of fatigue/malaise	−
Laboratory markers of thyrotoxicosis including decreased TSH and increased FT4-thyroid tests should be considered in all patients hospitalized due to COVID-19, especially in ICU patients	+
SAT is more frequently painless in COVID-19 patients and the presence of pain should not be treated as SAT criterion in this group, especially in hospitalized patients	−
As SAT may be the only manifestation of COVID-19, testing for SARS-CoV-2 infection should be considered in all patients with SAT diagnosed during the pandemic	−

Additional criteria (at least one should be met)	
Hard thyroid swelling	−
Pain and tenderness of the thyroid gland/lobe	+
Elevation of serum FT4 and suppression of TSH	+
Decreased radioiodine uptake	Unavailable
FNAB result typical for SAT	Unavailable

## Data Availability

The data used to support the findings of this study are available from the corresponding author upon request.
